# Correction: Impairment of cocaine-mediated behaviours in mice by clinically relevant Ras-ERK inhibitors

**DOI:** 10.7554/eLife.106301

**Published:** 2025-02-07

**Authors:** Alessandro Papale, Ilaria Maria Morella, Marzia Tina Indrigo, Rick E Bernardi, Livia Marrone, Francesca Marchisella, Andrea Brancale, Rainer Spanagel, Riccardo Brambilla, Stefania Fasano

**Keywords:** Mouse

Papale A, Morella IM, Indrigo MT, Bernardi RE, Marrone L, Marchisella F, Brancale A, Spanagel R, Brambilla R, Fasano S. 2016. Impairment of cocaine-mediated behaviours in mice by clinically relevant Ras-ERK inhibitors. *eLife* **5**:e17111. doi: 10.7554/eLife.17111.

Published 24 August 2016

We were notified by eLife journal and via PubPeer of three errors.

The first one is in Figure 3, where the “Scr RB1 cocaine” and “Scr RB3 cocaine” panels partially overlap. This unintentional duplication occurred during panel assembly. Since all histological analyses were performed in blind, captured images were assigned to numerical codes. During the preparation of the final images for the manuscript, treatments and conditions were made explicit and the selected representative images for publication were first duplicated in their original folders and then moved to “Final Folders”, containing only images for publication. During this process, an image from the original folder “Scr-RB1 cocaine” was accidentally duplicated twice and incorrectly placed into the Final Folder “Scramble RB3 Cocaine”. Consequently, both final folders named “Scr-RB1 cocaine” and “Scr-RB3 cocaine” contained the same image. To further complicate this matter, the images were cropped, making the duplication even harder to detect.

The corrected Figure 3 is shown here:

**Figure 1:**
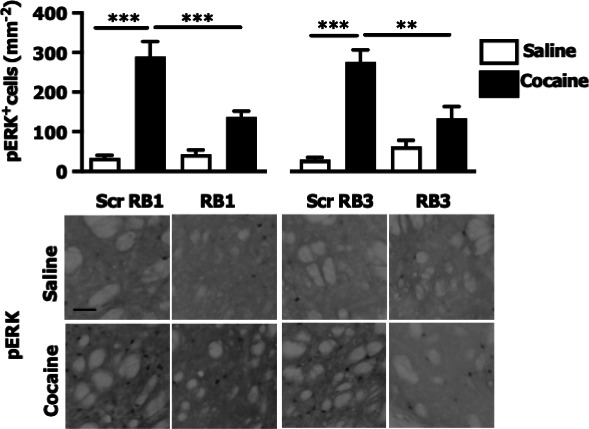


The originally published Figure 3 is shown for reference:

**Figure 2:**
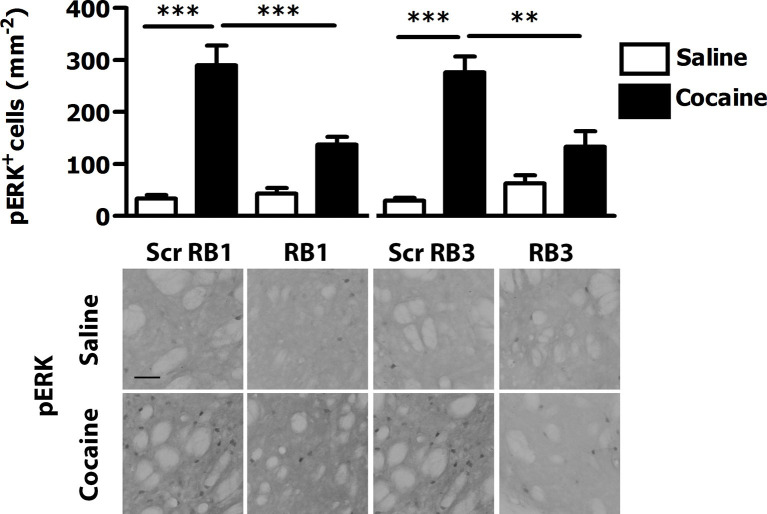


The second error is in Figure 2—figure supplement 1C, where "RB1 0.2 µM / Not stim" and "RB1 2 µM / Glu" panels partially overlap.

The third error is in Figure 2—figure supplement 1D, where “Scramble Glu” and “RB3 0.5 µM Glu” panels partially overlap. We believe that also these mistakes occurred during panel assembly, similarly to what happened in Figure 3. Unfortunately, due to the passage of time since the publication, we are unable to find the original uncropped images. Thus, Figure 2—figure supplement 1 has been removed from the paper.

Figure 2—figure supplement 1 which has now been removed from the published paper is shown for reference:

**Figure 3:**
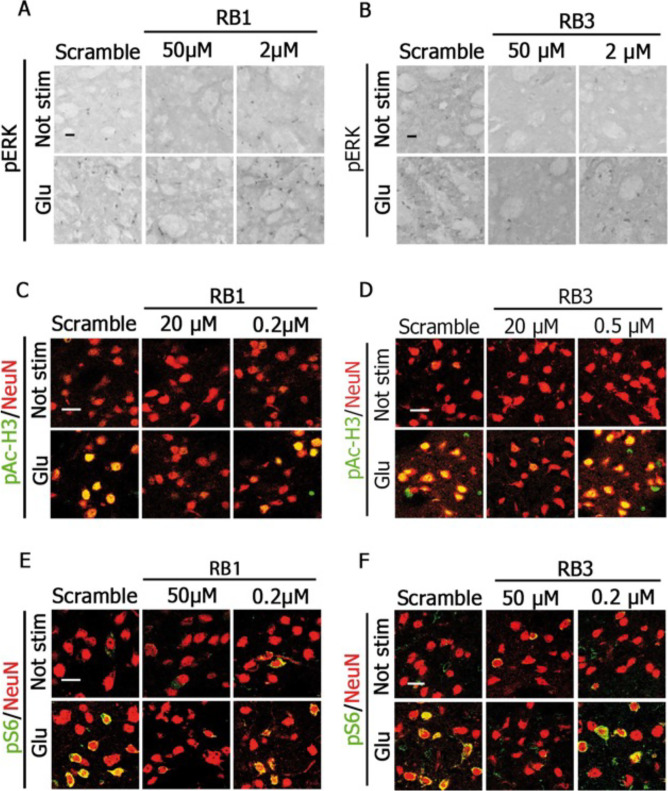


These mistakes do not affect to any extent the results, interpretation and conclusions of this work.

The article has been corrected accordingly.

